# Assessment of Discrimination, Bias, and Inclusion in a United States Hematology and Oncology Fellowship Program

**DOI:** 10.1001/jamanetworkopen.2021.33199

**Published:** 2021-11-08

**Authors:** Rahma M. Warsame, Gladys B. Asiedu, Ashok Kumbamu, Joselle Cook, Sharonne N. Hayes, Carrie A. Thompson, Timothy J. Hobday, Katharine A. R. Price

**Affiliations:** 1 Division of Hematology, Mayo Clinic, Rochester, Minnesota; 2 Robert D. and Patricia E. Kern Center for the Science of Health Care Delivery, Mayo Clinic, Rochester, Minnesota; 3 Division of Medical Oncology, Mayo Clinic, Rochester, Minnesota; 4 Department of Cardiovascular Diseases, Mayo Clinic, Rochester, Minnesota

## Abstract

**Importance:**

Medical trainees frequently experience discrimination. Understanding their experiences is essential to improving learning environments.

**Objective:**

To characterize trainee experiences of discrimination and inclusion to inform graduate medical education (GME) policies.

**Design, Setting, and Participants:**

This qualitative study used an anonymous telephone interview technique to gather data from hematology and oncology fellows. All current trainees and recent graduates were eligible. Interviews were conducted anonymously with interviewer and participant in separate locations and recorded and transcribed. Data were analyzed in an iterative process into major themes using a general inductive analysis approach. Demographic information was obtained via anonymous survey. Data collection and analysis were conducted from July 2018 to November 2019.

**Main Outcomes and Measures:**

Emergent themes illustrating bias and inclusion in a GME program.

**Results:**

Among 34 fellows and recent graduates who were approached for this study, 20 consented and 17 were interviewed. Of those interviewed, 10 were men, and the median (range) age was 32 (29-53) years. The racial and ethnic distribution included 6 Asian individuals, 2 Black individuals, 3 Hispanic individuals, 2 multiracial individuals, and 4 White individuals. All fellows reported having experienced and/or witnessed discriminatory behavior. The themes elucidated were (1) foreign fellows perceived as outsiders, (2) US citizens feeling alien at home, (3) gender role-typing, (4) perception of futility of reporting, (5) diversity and inclusion, and (6) coping strategies. The majority of reported biases were from patients. Only 1 trainee reported any incidents. Reasons for not reporting were difficulty characterizing discrimination and doubt action would occur. Participants reported that diversity of cotrainees, involvement in committees, and open discussions promoted inclusivity.

**Conclusions and Relevance:**

In this study, reports of discriminatory behavior toward trainees were common. The anonymous hotline methodology cultivated a safe environment for candid discussions. These findings suggest that GME programs should assess their learning climate regarding bias and inclusivity anonymously and develop processes to support trainees.

## Key Points

**Question:**

What are the experiences of trainees with respect to discrimination, bias, and inclusion during hematology and oncology fellowship?

**Findings:**

In this qualitative study of anonymous hotline interviews with 17 fellows, 100% of the fellows reported experiencing or witnessing discriminatory behavior, mostly from patients; a novel theme was alien at home, referring to US citizens from racial or ethnic minority groups treated as other or foreign. Reporting was infrequent due to belief of futility; and diversity of the fellows in the program contributed a sense of inclusion.

**Meaning:**

These findings suggest that hematology and oncology trainees need better protection from discrimination and processes for reporting witnessing of discrimination.

## Teaser

This qualitative study investigates the experiences of discrimination and inclusion among trainees and recent graduates of a US hematology and oncology fellowship program.

## Introduction

The social justice events of recent years in the United States, including the MeToo and Black Lives Matter movements, have underscored the presence of systemic racism and sexism. Change is imperative in medicine with woeful disparities in racial and ethnic minority groups, lack of diversity among physicians, and limits to the success of women and minority groups.^1,2^ Moreover, the literature demonstrates that underrepresented minority groups and women^3^ in medicine experience disproportionate discrimination and bias that affects their careers from medical school to faculty.^4,5^ The clinical learning environment during training can be affected by discrimination and by factors that cultivate inclusivity and safety. Graduate medical education (GME) programs strive to create a safe and welcoming learning environment as it can affect patient care, education quality, and trainee well-being. Understanding the nature, extent, and impact of discrimination toward trainees and elements of inclusivity require in-depth discussions that may be challenging as many trainees fear reporting owing to concerns about retaliation, investigation, and adverse effect on future job prospects.^6^ Further complicating matters are the mandatory reporting requirements of gender- or personal characteristics–based discrimination under Title VII and IX laws, which subject learners to potential investigation of reported events.

A detailed understanding of trainee experience with discrimination and inclusion is critical to allow the development of evidence-based strategies to support trainees, strengthen inclusivity, and improve the learning environment. Prior studies investigating the prevalence of discrimination among medical trainees have been conducted using various methodologies. These studies do not explicitly outline whether Title VII and IX laws were clearly addressed to participants, or any reporting of data obtained.^5,6,7,8,9,10,11,12,13,14,15^ Also, there is a dearth of data on what fosters inclusivity in GME programs. To fill this gap in the literature, we conducted a pilot study of our hematology and oncology fellows using a novel anonymous hotline interview method (coined by the study team) to obtain rich data of trainee experience of bias, discrimination, and inclusion to inform program and GME policies that also considered Title VII and IX laws protecting trainees. Anonymous telephone interviews (hotline approach) have been used by various government and nongovernment agencies in areas of law enforcement and employee concerns. In the health sector it has been used by the public to anonymously report sensitive topics including abuse and mental health issues, but, to our knowledge, its use in qualitative research has not been explored. In our study, we used this interview technique for research to gather data from participants who experienced or witnessed any form of discrimination or bias and to understand components of inclusion. This approach was valuable to understand trainees’ experiences anonymously so that the information gathered did not pose a challenge to our institution’s compliance policies.

## Methods

### Study Design

This study developed an anonymous hotline interview technique to characterize discrimination and inclusivity experiences of hematology and oncology fellows while protecting their identity. The principal investigators (PIs) partnered with the institution’s Compliance Office to ensure the study did not violate Title IX or Title VII. An interview guide was developed for the in-depth, semistructured interviews addressing the themes of discrimination, bias, and inclusion, and allowed deeper exploration of the type and cause of the discrimination, the perpetrator of the discrimination, trainee reaction to the events, and elements of inclusivity (eAppendix 1 in the Supplement). Deidentified transcripts were reviewed by the PIs to screen for reportable events (eAppendix 2 in the Supplement). Reportable events were reviewed and reported to the Compliance Office but could not be linked to individual participants. The study was approved by the Mayo Clinic institutional review board.

### Study Participants and Recruitment

All current hematology and oncology fellows and recent graduates on staff (within 6 months) were eligible and invited to participate via email. Participants reviewed and signed a consent form privately with one of the PIs to allow for questions and explanation of the study methods, which included mandatory reporting of any egregious events or recurrent patterns despite anonymity. To decrease connection between time of consent and order of interviews, written consent was obtained from all participants before any interviews were conducted. Weekly emails with hotline availability were sent to all participants. The Figure shows recruitment and data gathering method.

**Figure.**
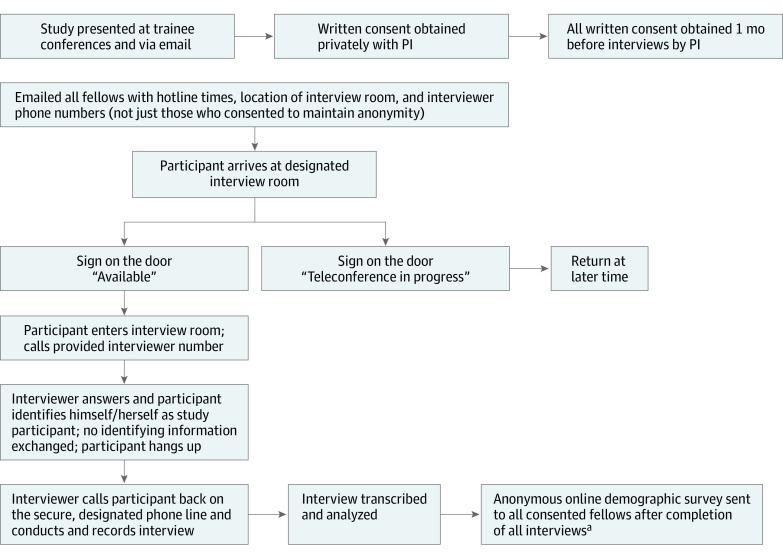
Recruitment and Data Gathering Methods ^a^Fellows who consented but did not complete the interview were not asked to fill out the survey.

### Data Collection

Interviews were conducted between July 1, 2018, and November 15, 2018. All interviews were recorded over the phone using standard recording devices, with both interviewer and participant in separate locations on campus. Using the hotline technique, participants made calls from a designated private room to the interviewers (Figure). All interviews were conducted by 2 team members who had no supervisory role over participants, did not work in the department, and did not know the fellows so voice recognition was not possible. Furthermore, no identifying information was exchanged during interviews, so participants and interviewers were unaware of who they were talking to. All interviews followed a semistructured interview guide (eAppendix 1 in the Supplement) which provided topic areas with probes for a systematic and comprehensive interview.^16^ All interviews were recorded, transcribed verbatim, and deidentified for analysis. Deidentified transcripts were stored electronically in a secure shared folder. Original audio recordings were destroyed once transcriptions were complete. With the aim of achieving data comprehensiveness rather than saturation, we chose to fully interview all respondents who agreed to participate. All demographic information was obtained via an anonymous online survey to define the study population but was not linked to the transcripts.

### Data Analysis

All transcripts were entered into the qualitative analysis software (Nvivo 11 [QSR International Pty Ltd]) and analyzed using a general inductive analysis approach.^17^ Initially 2 coders did an open reading of 4 randomly selected transcripts to identify some major emerging themes. Based on initial reading of transcripts (emerging codes), literature review (a priori codes), and study team discussions and reflexivity, a codebook was developed with definitions. All transcripts were coded independently by 2 qualitative researchers. Consensus was achieved through discussion with both PIs and coders in scheduled data analysis meetings. Based on the code book, emerging themes were identified. Major themes were further refined and synthesized into 6 categories, with representative quotes supporting each theme. Our team has diverse backgrounds in oncology, hematology, sociology, family science, and health services, which enabled analyst and investigator triangulation, via reflexivity, allowing for confirmation of findings across study team and enhancing credibility of findings.

## Results

### Study Participants

All 29 current hematology and oncology fellows and 5 newly graduated faculty who remained on staff were approached. Among the 34 approached, 20 (59%) signed written consent (15 current fellows, 5 newly graduated). Seventeen (17/20; 85% of consented) completed interviews. Of these 17 study participants, 9 (53%) were Asian or Asian American, 2 (12%) were Black or African American, 3 (18%) were Hispanic or Latino, and 2 (12%) were multiracial; 10 (59%) were male; and the median (range) age was 32 (29-53) years (Table 1). The median (range) interview length was 30.6 (12.4-59.4) minutes.

**Table 1.** Participant Demographics

Characteristic	Participants, No. (%)
No.	17
Age, median (range), y	32 (29-53)
Sex	
Female	7 (41)
Male	10 (59)
Race	
Asian or Asian American	9 (53)
Black or African American	2 (12)
White or Caucasian	4 (23)
More than 1 race	2 (12)
Ethnicity	
Hispanic or Latino	3 (18)
Marital status	
Married	13 (76)
Single	4 (24)
Religion	
Agnostic	1 (6)
Catholic	3 (18)
Christian	5 (29)
Hindu	5 (29)
None	3 (18)
Speak English with accent	
Yes	6 (35)
No	11 (65)
Years of experience, median (range)^a^	6 (3.5-9)

^a^ Years of experience since graduating medical school.

We identified 6 major themes (Table 2) that reflected discriminatory events experienced or witnessed by participants, the majority of which came from patients not employees (faculty, trainees, allied health, and other employees). Most of the incidents described were interpreted by our study team as microaggressions, whereas macroagressions were rarer events (eAppendix 3 in the Supplement).^18^ Six trainees were aware of policies for reporting patient misconduct or discrimination; only one ever reported an incident. The major themes included (1) foreigner or perceived as other, (2) misidentification or alien at home, (3) gender role typing, (4) minimization and futility of reporting, (5) diversity and inclusion, and (6) coping. The reported impact of the discriminatory events included personal anguish and motivation to improve communication. The identified themes are described as follows, with representative quotes illustrated in Table 2.

**Table 2.** Emerging Themes and Associated Quotes

Theme	Quotes
Theme 1: foreign or perceived as other	
Feeling of being other or a foreigner in their professional environment.	“I was fired by a patient because I have an accent…it’s the same feeling like you don’t belong [here], and it it’s hard because we all went to medical school…I called him [patient] to see how he was doing, and he’s like, I don’t want a doctor that doesn’t know how to speak English. I want an American doctor. I don’t want to see you anymore, and he hung up on me.” [Participant 14]
	“I will say that there is no culture for appreciation of being different. There is only appreciation for assimilation.” [Participant 9]
	“You enter a room, and see the patient’s face actually fall—the only things is that you’re not sure if it’s because you are fellow or if it’s because you’re not white.” [Participant 12]
These encounters were described as coming from patients and experienced by both fellows, residents, and consultants irrespective of the number of years of experience in their practice.	“I know sometimes that happens more to junior people, but this was very shocking because it happened to such a senior person too … I felt angry for that attending because [he had] taken like 20 y to make himself the world expert in this and then he also, again, has to face the same thing.” [Participant 2]
Theme 2: misidentification and alien at home	
US-born trainees made to feel like outsiders by patients and employees.	“I am of Indian heritage, and so a lot of my patients will ask me questions about, where are you from? And I actually grew up and was born in Wyoming and then they follow it up with usually like ‘No, where are you from’?” [Participant 9]
	“I am Asian person and often patients ask, ‘Where are you from?’ And I say I was born in xxx. And they say, ‘Oh well, but before then’ [laughs] it’s like well before I was born?…Sometimes I feel a bit of pressure to be more American and kind of prove that I am just as a American as they are.” [Participant 15]
	“I want an American doctor. I don’t want to see you anymore…” [Participant 14]
	“I was told [by attending] ‘Well you didn’t grow up here and you don’t know that. That is only for people who grew up here.’ And I was born here on top of that!” [Participant 14]
	“When I was interviewing for this fellowship, and one of my attending that I knew fairly well asked during my interview what my visa status was…this is my citizenship that I was born with, just like that attending was.” [Participant 9]
Theme 3: gender role typing	
Participants reported differential treatment and inappropriate comments toward female trainees by patients and employees. Some of these experiences were witnessed by male trainees who also reported seeing how their female colleagues were treated differently. Experiences were around being disregarded, not acknowledging their credentialing, having higher expectations for female trainees than male trainees, and being asked questions about female trainees’ personal matters.	“I tend to have my patients calling me by my first name, I’m happy to be on a personal level with my patients but I also want the same amount of respect that they would give one of my male colleagues.” [Participant 4]
	[Patient told female physician] “I don’t like women doctors because you may not know what you are doing.” [Participant 14]
	“‘Oh what happened? Did you get divorced’ referring to when I am not wearing my wedding ring.” [Participant 14]
	“I think that’s difficult for some of my female partners and colleagues to get the credibility, they have a higher threshold to establish credibility…not trust their opinion as well until someone else [male colleagues] reaffirms it.” [Participant 3]
	“I think subconscious they [nurses] tend to treat male physicians better, to be honest.” [Participant 3]
	“I have had a few nurses that have looked to my male resident and I am the fellow…looking for advice instead of acknowledging me who is the one running the team.” [Participant 14]
	“I worked with an attending for a week and he barely addressed me or made eye contact with me and would only make eye contact with my male co-fellow.” [Participant 12]
	“I’m interviewing for jobs...and it always comes up. Do you have children? Like maybe they are asking in an innocuous manner, but I feel like people always worry. Is this person going to take maternity leave and be less available for work?” [Participant 2]
Theme 4: minimization and futility of reporting	
Normalization of discriminatory behavior such that reporting is not pursued, or belief of lack of accountability.	“I am afraid to report these things because, there’s gonna be repercussions. There’s no way it’s gonna be anonymous…I just have to toughen up and, you know get used [to it].” [Participant 12]
	“The tough part is I don’t know how much you can do to affect, a patient’s probably longstanding beliefs. You can’t technically prove it, unless they are explicitly saying something.” [Participant 3]
	“I think just a lot of talking and no actions.” [Participant 14]
Participants raise a variety of concerns about reporting their experiences—micro-invalidation, lack of consequences and trust in the reporting systems consequences and the nature of those experiences that involve subtle and nuanced behaviors are difficult to prove.	“I did not discuss with anybody…I mean no, none of the consultants or the mentors because, you know, I thought that they wouldn’t be able to do anything about it and I couldn’t get more information, and so I didn’t even think that they would do anything about it anyway.” [Participant 11]
Theme 5: diversity and Inclusion	
Aspect of participants’ fellowship experience or other’s behavior, policies and procedures and systems in place that makes them feel welcome or gives them a sense of belonging.	“The best part of the fellowship is that you have a lot of diversity.” [Participant 17]
	“Since there is so much diversity that makes me feel welcome.” [Participant 15]
	“When I came in to see that there was someone else that looked like me in the class that was a little reassuring.” [Participant 7]
	“Fellows are included in all, institutional committees, which is unique. There’s a fellow representative… This, kind of makes you feel that your voice is heard.” [Participant 2]
	“The humanities sessions…find them very helpful …because it’s just an opportunity to talk about issues that you face going through training, and several where we’ve talked a lot about bias and discrimination.” [Participant 4]
Seeing other trainees that looked like participants, availability of inclusive programing and activities, having the opportunities to discuss, debrief and be involved in the program were few experiences that made participants feel included in their program.	“The fellowship program meets with all the fellows once a month as a group to discuss issues. So if you have some issues that you need to bring up, you can—and it’s a pretty nonthreatening environment” [Participant 2]
Theme 6: Coping	
What participants do to help them cope with negative interactions or experiences that happen at their work; coping mechanism after a discriminatory event was debriefing with friends/family/co-fellows and focusing on the abundant positive patient experiences.	“Initially it didn’t really bother me, like I would just kind of blow it off. But it actually has started to bother more and more because I think as I’ve gotten older…” [Participant 9]
	“I went home and cried, and I am still dealing with it…the worst part I was born in this country.” [Participant 14]
	“I cannot just stay there, so I found a very nice humorous way to make him stop talking.” [Participant 1]
	“And I just don’t talk about it, unless it’s with someone of my own ethnicity who is also going through the very same thing and that’s—there is not a lot of that so it’s not something that I talk about often, and I feel uncomfortable talking about, uh, race discrimination, um, in general.”
	“I guess crying helps. And I- I don’t- I don’t really feel like have somebody who- who I can go in my leadership in the program and just talk. I don’t feel confident. So I talk to my husband, or I talk to my classmates. I think that’s my coping mechanism.” [Participant 14]
	“I want to be able to shake these things off, in order to, you know, continue to taking- take good care of the patient and, you know, get along with my day that I- I'm able to kind of compartmentalize it and just say, Let’s go forward and, you know, I'll vent about it later.” [Participant 4]

#### Theme 1: Foreigner or Perceived as Other

This theme was the feeling of being other or a foreigner in their professional environment. Trainees described that some patients perceived the presence of an accent as a sign of an inferior physician. There was a range of ways this manifested from outright firing by patients to asking subtle questions of fellows’ heritage. Many fellows found these events disheartening but assumed these experiences would be limited to their training; however, there were instances where participants witnessed the same behavior toward their staff. Some fellows reported suspicion that a patient question or behavior was motivated by underlying bias toward the trainee’s race, ethnicity, and/or nationality. Participants felt that questions about heritage were not simply innocent curiosity but rather an attempt by patients to determine if the trainee is one of us. These experiences created psychological fatigue as participants tried to understand why patients and employees displayed these behaviors. Discriminatory or biased behavior related to being foreign-born was predominantly from patients and seldom reported about employees or staff.

#### Theme 2: Misidentification and Alien at Home

Trainees who were US citizens and identified as Asian or Asian American, Black or African American, or more than 1 race reported being misidentified as foreign because they did not meet preconceived notions of what constitutes American. These experiences ranged from overt requests for “an American physician” to indirect exchanges that belittled fellows. The inquiries into a fellow’s race, ethnicity, or nationality and questions of heritage for US-born trainees caused emotional distress by raising concern about their legitimacy in their nation and questioning their sense of belonging. These implicit or explicit biases of what an American looks like were perpetrated by employees as well as patients. The frequent experiences of feeling alien in one’s nation negatively impacted trainee sense of belonging in the program and institution.

#### Theme 3: Gender Role Typing

All reports of gender discrimination were directed toward women and described by both male (witnessed) and female (experienced and/or witnessed) trainees. There was a range of reported events from patients toward female trainees that included refusal to see a female physician, inappropriate comments about appearance or marital status, and discounting credibility or expertise. Female trainees frequently reported being mistaken for the nurse or other allied health staff. Some male colleagues commented on how unlikely this is to happen to a male trainee.

Gender discrimination from employees toward female trainees was different than that from patients. With respect to employees, trainees reported negative interactions between nurses and female physicians, and differential treatment and teaching by faculty compared with male trainees. The cumulative effect of these actions was to undermine the learning experience of the female trainees compared with their male counterparts. Female trainees reported responding to incidents by self-promotion, asserting their competency, and informing patients and employees of their knowledge and capabilities. Female trainees exclusively reported concern for how starting a family would impact future employability.

#### Theme 4: Minimization and Futility of Reporting

Despite numerous examples of discriminatory behavior toward trainees, only one fellow ever reported an incident to program leadership and no one reported to the Compliance Office or human resources. Most trainees were unaware of policies to protect them, and how to report. Concerns about reporting included jeopardizing future employability, risk of retaliation, and challenges reporting experiences that could be perceived as subjective and difficult to prove. Even if a trainee considered reporting, they often did not due to a perception of futility. Explicit patient behavior, such as firing a trainee, was usually addressed in the moment with the supervising physician, but it is unknown whether the faculty formally reported the incidents. Often the faculty assumed care of such patient without any follow-up to the trainee.

#### Theme 5: Diversity and Inclusion

The predominant theme that emerged from questions about inclusion was that diversity itself is important to cultivate inclusion. The fellows frequently reported that the substantial diversity of the fellowship program inherently creates an inclusive environment. Furthermore, the presence of diverse trainees demonstrates the commitment to diversity and to creating a welcome learning environment. Creating safe spaces for discussion was critical to foster inclusion. The participants cited the fellows’ workroom, fellow-only meetings, and participation in a faculty-run voluntary hematology and oncology fellowship humanities session to address topics unique to oncology, including the ethics and existential questions, as key to making them feel heard and welcomed. Representation on institutional committees also made trainees feel that their voice mattered on issues that pertain to hematology and oncology fellowship research, practice, and education.

#### Theme 6: Coping Strategies

This theme described what participants did to help them cope with negative interactions or experiences that occurred during training. The most common coping mechanism was debriefing with friends, family, cofellows, and others with concordant identities and focusing on the abundant positive patient experiences. Participants also coped by ignoring the negative experiences and focusing on the meaningful positive experiences.

## Discussion

To understand the experiences of hematology and oncology fellows in our program, we conducted a qualitative study to explore nuances of their experiences with both discrimination and inclusion. We describe our methodology for safely evaluating trainee experiences through anonymous hotline phone interviews. This study highlights the pervasive nature of bias and discrimination against trainees with 100% of those interviewed reporting experienced or witnessed discriminatory behavior.

The current sociopolitical environment has highlighted systemic racism and the impact it has on medicine and medical education. A recent cross-sectional study found that among 262 surgical US residency programs (n = 7409 residents), 32% reported discrimination due to self-identified gender, 16% reported racial discrimination, and 10% reported sexual harrassment.^19^ There have been calls to better protect physicians (and trainees) through curriculum development on handling patient bias and focused training, such as bystander training, to empower individuals to intervene, and faculty development and debriefings. Unfortunately, these recommended measures have been slow to implement.^20,21,22,23,24^ We describe major recurring themes of bias and discrimination experienced by fellows which ranged from explicit macroaggressions to subtle incidents. Most incidents were patient-related, and there was a sense of futility in reporting. Notably in our study, trainees did not raise any issues regarding discrimination based on religion. Program diversity created a sense of inclusion. We found strategies to enhance inclusivity were intentional recruitment of diverse trainees, forums for trainees’ perspectives to be heard, and the creation of safe spaces, as suggested by American College of GME. This was done via institutional committee involvement, regular meetings focused on addressing trainee concerns, and informal gatherings dedicated to discussing unique aspects of training that included ethics, racism, sexism, and suffering.^25^

Several studies report the challenges for physicians and trainees from minoritized groups and those who are considered foreign.^4,5^ Yet there is a dearth of evidence documenting the challenge for the US citizen who feels alien at home. Our study highlights that this was a common detrimental issue that should be recognized particularly given that our national population continues to diversify. Our study gives further credence to the existing body of literature outlining the disparities that women physicians face.^3,13,26,27,28^ Our findings also support efforts to increase physician diversity as this is beneficial in fostering a culture of inclusion. Our study underscores the culture of silence surrounding bias in medicine and widespread fear or skepticism of reporting. Incidents went almost universally unreported; more concerning still was the belief that reporting has no potential for improvement or change.^4,29,30^ The reluctance of trainees to report abusive or inappropriate behavior may be attributed to the fear of retaliation and jeopardizing their future career but emphasizes the lack of confidence in programs’ ability to protect and act for trainees. Medical institutions must create processes for reporting incidents of discrimination and teach faculty debriefing strategies to support trainees.^30,31,32^

### Strengths and Limitations

This study had some strengths and limitations. We used anonymous hotline interviews as a research methodology to elicit physician trainee experiences with discrimination, bias, and inclusion. We demonstrated that this approach is feasible and effective, with 85% of participants completing the phone interview and all participants candidly describing sensitive information. The anonymous interview technique was not disruptive to data gathering for research. Despite concern that the technique could create challenges for study participants, the approach created confidentiality and ease of participation. The hotline was not disruptive; it reinforced commitment to trainee safety, privacy, and freedom to speak. The benefit of this method was the anonymity of participants that facilitated candid testimony free from consequence, while allowing for compulsory reporting under federal law. Several studies including one of 548 medical students and one of 1773 residents utilized surveys or questionnaires where it was not clear whether it was or was not anonymous.^33,34,35^ A recent qualitative survey of physicians and trainees on patient bias was conducted by convenience sample focus group assessment; although clear themes emerged, that approach prevented anonymity and therein the breadth of perspective.^20^ In contrast, our study methodology permitted any eligible trainee to use the anonymous hotline to describe their experience. Importantly, our anonymous hotline approach is the first to describe methods that deliberately considered institutional compliance policies in the study development process. This format could be critical for future study of sensitive topics, particularly vulnerable populations where power differential is not in their favor, as it mitigates distress to participants regarding fear of retaliation and cultivates a safe space for candid reporting yet allows institutions to investigate pertinent topics and/or recognize patterns.

The study is limited by its focus on one subspecialty and lack of documentation of why some fellows chose not to participate, potentially limiting generalizability. However, rich data can be elicited from small populations. The anonymous hotline methodology, while protecting the participants and allowing for rich narrative data on sensitive issues, did pose some challenges as specific appointment times were not able to be scheduled and some individuals who consented did not interview. Although the methodology did lack nonverbal communication, it did not appear to inhibit the acquisition of sensitive testimony.

## Conclusions

This qualitative study found that discriminatory behavior toward trainees continues to be prevalent, frequently coming more from patients than staff, and supports the idea that diversity and inclusion are synergistic. It also describes an underreported experience of medical trainees who are US citizens from underrepresented racial or ethnic groups. The anonymous hotline approach is feasible and effective to explore sensitive topics and scalable to various geographic locations and different medical specialties. Study results were shared with programmatic leadership and prompted program wide training for staff to strengthen their skills to address discriminatory incidents and better support trainees who are targeted. These findings suggest that it is imperative for GME programs to create a supportive learning environment to protect trainees and ensure equity in the educational experience.

## 

## Notes

Supplement. **eAppendix 1.** Interview Guide **eAppendix 2.** Definition of a Reportable Event **eAppendix 3.** Definitions
