# Supplementary material for: Assessment of Discrimination, Bias, and Inclusion in a United States Hematology and Oncology Fellowship Program
Source: JAMA Netw Open. 2021 Nov 8;4(11):e2133199. doi: 10.1001/jamanetworkopen.2021.33199 (PMC8576584; doi:10.1001/jamanetworkopen.2021.33199)
Supplement: Supplement. — eAppendix 1. Interview Guide eAppendix 2. Definition of a Reportable Event eAppendix 3. Definitions [file jamanetwopen-e2133199-s001.pdf]

Supplemental Online Content

Warsame RM, Asiedu GB, Kumbamu A, et al. Assessment of discrimination, bias, and inclusion in a United States hematology and oncology fellowship program. *JAMA Netw Open.* 2021;4(11):e2133199. doi:10.1001/jamanetworkopen.2021.33199

eAppendix 1. Interview Guide

eAppendix 2. Definition of a Reportable Event

eAppendix 3. Definitions

This supplemental material has been provided by the authors to give readers additional information about their work.

© 2021 Warsame RM et al. *JAMA Network Open.*

eAppendix 1 - Interview Guide (45-60mins)

A. **<u>Establishing a common ground of Terminology</u>**
1. First I'd like you to explain to me, in your own words, what discrimination, bias or harassment means?

B. **<u>General clinical experiences (icebreaker).</u>**
1. Let's start by talking in general about your clinical experiences in your training as hematology/oncology fellow.
2. How does your everyday life look like in your training practice?

A. **<u>Establishing evidence of discrimination experience from patients</u>**
3. Have you experienced bias/discrimination from a patient during your fellowship? **<u>Alternatively:</u>** Do you feel as though you have been treated differently than others (based on your identity: race, culture, religion, religion, national origin, accent, marital status, color, creed, sexual orientation, gender identity or expression, disability (physical and mental), by a patient?
    a. **<u>If Yes:</u>**
    Describe your experience to me
    Why do you think you were treated differently?
    Did you report it or shared it with anyone?
    Could you describe how it was handled by the individual to whom you reported?
    What are the most important ways that this/these experience/s impact your life? (*Did the experience change anything about your practice? Did the experience change your perception of yourself? Did the experience adversely affect your education?)*
    b. **<u>If No:</u>**
    Imagine you heard of a colleague who had experienced discrimination/harassment, what would you do?

4. Have you witnessed bias/discrimination towards Mayo Clinic learners (trainees, fellows) from a patient? If Yes explain *(what, how and why)*
5. During your training program did you witnessed bias/discrimination towards any Mayo Clinic employee from a patient? If Yes explain. *(what, how and why)*

B. **<u>Establishing evidence of 'other' discrimination practices</u>**

© 2021 Warsame RM et al. *JAMA Network Open.*

6. Now let's talk in general about discrimination and bias from Mayo clinic employees during your fellowship training. Have you experienced discrimination/bias (assault, harassment) from Mayo Clinic employees (staff, nurse, clinical team etc)?
   Alternatively: Do you feel as though you have been treated differently than others based on your gender, religion by Mayo clinic employees (staff, nurse, clinical team etc)? What are your experiences?
   a. **If Yes:**
      Describe your experience to me
      Why do you think you were treated differently (why do you think you experienced this)?
      Did you report it or shared it with anyone? What happened?
   Could you describe how it was handled by the individual to whom you reported?
   What are the most important ways that this/these experience/s impacted your life? *(Did the experience change anything about your practice? Did the experience change your perception of yourself? Did the experience adversely affect your education?)*
   b. **If No:**
   Imagine you heard of a colleague who had experienced discrimination/harassment from other Mayo Clinic Employees, what would you do?
7. Have you experienced discrimination/bias from Mayo Clinic learners/faculty?
   Alternatively: Do you feel as though you have been treated differently than others by Mayo clinic learners/faculty?
   a. **If Yes:**
      Describe your experience to me
      Why do you think you were treated differently (why do you think you experienced this)?
      Did you report it or shared it with anyone?
      Could you describe how it was handled by the individual to whom you reported?
   What are the most important ways that this/these experience/s impacted your life? *(Did the experience change anything about your practice? Did the experience change your perception of yourself? Did the experience adversely affect your education?)*
   b. **If No:**

   Have you ever shared these experiences with anyone? Please explain.
   Imagine you heard of a colleague who had experienced discrimination/harassment from other Mayo Clinic faculty/learners, what would you do?

C. **Establishing Evidence of Inclusivity.**

8. I'd like to shift our conversations to your experiences around inclusivity. By inclusion experiences we mean aspect of your fellowship or other's behavior, policies and procedures and systems in place that makes you feel welcome and gives you a sense of

© 2021 Warsame RM et al. *JAMA Network Open.*

belonging. Are there aspects of your fellowship/training experiences that make you feel welcome and give you a sense of belongingness? Please explain
   a. Do you feel a sense of belonging within the fellowship?
      i. If so, what people, actions, or practices contribute to the feeling of belonging?
      ii. If not, what people, actions, or practices have made you feel excluded?

9. How would you assess your fellowship program in terms of its openness and diversity?
   a. Describe how you are able to speak your mind without fear of retaliation and/or negative consequences?

10. How would you assess the environment of the Division of Hematology and Department of Oncology **(in terms of its inclusive nature)**?
    a. Can you share some of the ways that the Division and Department create inclusive environment for all regardless of race, ethnicity, gender, sexuality disability, age, accent, and other differences?
    b. Describe some of the efforts that are made to make everyone feel welcome?
11. Were there certain actions, policies, or gestures that made you feel welcome at Mayo when you started your training program? Please explain.
12. Are there certain aspects of your daily work life that are positive and make you feel happy and/or included?

Coping with Negative Experiences

13. Now that you have described your experiences of discrimination and bias, can you share with me how you cope with the experiences that you shared with me today?
    a. What do you do to help you cope with negative interactions or experiences that happen at work?
14. Are there specific individuals who have been important to help you cope with adverse events?

Recommendations and Suggestion for Training Improvement

15. How can the institution/department improve the process of welcoming trainees and making them feel included?

© 2021 Warsame RM et al. *JAMA Network Open.*

eAppendix 2 - Definition of a Reportable Event

If an egregious or criminal event is reported: The PIs will review all of the de-identified phone interview transcripts. If there is an event that is deemed reportable (see list below), the transcripts will be shared with the Compliance Office. *However, there will be no way to link the identity of any given transcript with a specific individual, so all participants will be protected from a targeted investigation, allowing them to speak freely and safely.* Such events could trigger a more generalized investigation into the issues, but the individual participants will not be able to be identified.

Definition of a Reportable Event:

1. Allegations of exceedingly offensive, harassing and/or vulgar language or jokes directed at an individual (patient, staff, trainee, etc.).
2. Allegations of perceived inappropriate touch.
3. Allegations of systematic or habitual discrimination against staff or trainees.
4. Allegations of perceived harassment based on an abuse of power.
5. Allegations of sexual assault (rape, molestation, etc.).
6. Allegations of any other egregious acts (at the discretion of the investigators).
7. Allegations where there is a pattern of misconduct alleged against an employee or trainee either through multiple instances of misconduct or multiple complainants.
8. Any behavior that could be considered criminal in nature.

© 2021 Warsame RM et al. *JAMA Network Open.*

eAppendix 3 - Definitions

Microaggressions: Defined as commonplace daily verbal, behavioral or environmental indignities whether intentional or unintentional that communicates negative prejudicial slights and insults toward any group or individual, particularly those who are marginalized

Macroaggressions: Obvious and intentional aggression towards another marginalized group or individual

© 2021 Warsame RM et al. *JAMA Network Open.*
